# Prognostic value of histogram analysis in advanced non-small cell lung cancer: a radiomic study

**DOI:** 10.18632/oncotarget.22316

**Published:** 2017-11-06

**Authors:** Bluthgen Maria Virginia, Faivre Laura, Rosellini Silvia, Ferrara Roberto, Facchinetti Francesco, Haspinger Eva, Ferte Charles, Ammari Samy, Michiels Stefan, Soria Jean-Charles, Caramella Caroline, Besse Benjamin

**Affiliations:** ^1^ Department of Cancer Medicine, Gustave Roussy Cancer Center, 94805 Villejuif, France; ^2^ Department of Biostatistics and Epidemiology, Gustave Roussy Cancer Center, 94805 Villejuif, France; ^3^ Department of Radiology, Gustave Roussy Cancer Center, 94805 Villejuif, France; ^4^ INSERM U1018, CESP, Université Paris-Sud, Université Paris-Saclay, Villejuif, France; ^5^ Université Paris-Sud, 91400 Orsay, France

**Keywords:** non-small cell lung cancer, advance, histogram analysis, texture, prognosis

## Abstract

**Introduction:**

Quantitative assessment of heterogeneity by histogram analysis (HA) of tumor images can potentially provide a non-invasive prognostic biomarker. We assessed the prognostic value of HA and evaluated a correlation with molecular signature.

**Results:**

CT scans performed between July 2009 and January 2015 from 692 patients were reviewed. HA was performed on scans from 313 patients in the training dataset and 108 in the validation dataset. Median follow-up were 33.7 months [range: 1.7 - 65.5] and 29 months [range: 1.1 - 35.6] with a median overall survival (OS) of 11.7 months [95%CI: 10.7 - 13.1] and 9.5 months [95%CI: 7.9 - 12.7] respectively. Primary mass entropy in coarse texture with spatial filter 3.3 was prognostic for OS in a multivariate Cox analysis (HR: 1.3 [95%CI: 1.1 - 1.5], *p*=0.001). Results were not reproduced in our validation set and no correlation with molecular signature was identified.

**Materials and Methods:**

HA using filtration-histogram method was applied to the region of interest on the primary tumor in enhanced-CT acquired as diagnostic/staging routine, from a cohort of patients with advanced non-small cell lung cancer (NSCLC) treated with platinum-based chemotherapy. The resultants parameters were prospectively applied to a validation dataset. CT scans, clinical and molecular data were retrospectively collected. Cox proportional hazard models were used for survival analysis and Wilcoxon test for correlations.

**Conclusion:**

Primary mass entropy was significantly associated with survival in the training set but was not validated in the validation cohort, raising doubt over the reliability of published data from small cohorts.

## INTRODUCTION

Tumors are composed of different populations of clonal and sub-clonal cells with distinct genotypic and phenotypic features, a phenomenon termed primary intra-tumor heterogeneity [1, 2]. In the “branched evolution theory”, an early somatic mutation common to all cancer cells is followed by subsequent late sub-clonal mutations. Intra-tumor heterogeneity is known to contribute to disease progression, poor clinical outcome and evolution of resistant clonal cells during treatment [3] in several tumor types including lung cancers [4–6]. Next generation sequencing of multiple biopsies within the same tumor elegantly illustrates this concept, however invasive and repeated tissue sampling to map a tumor is unrealistic in daily practice for patients. Measurement of spatial heterogeneity through evaluation of image pixel distribution has received increasing attention in the last decade. Image analysis has the potential to provide a non-invasive assessment of tumor heterogeneity. Among the available options, histogram analysis (HA) represents an emerging practical technique to quantify tumor heterogeneity from a computed tomography (CT) image [7]. It refers to a collection of mathematical methodologies used to evaluate the gray-level intensity, and distribution of the pixel values within the tumor image. The resulting histogram of pixel distribution is quantified by standard descriptors or parameters (mean, standard deviation, skewness and kurtosis) as a measure of intralesional heterogeneity that can be correlated with clinical and histological variables providing potential prognostic and predictive biomarkers [8, 9].

Assessment of tumor heterogeneity through histogram analysis represents an interesting non-invasive alternative approach and has the potential to provide a prognostic tool to assist oncologists when choosing therapeutic approaches. In non-small cell lung cancer (NSCLC), treatment decisions are generally based on molecular categorization, however adequate material for analysis remains an issue. Various histogram-based parameters have been proposed as potential prognostic tools in light of their correlation with histology [10], molecular profile [11, 12], response to treatment [13] and survival [14–16]. However, in these studies, populations were relatively modest in size, were heterogeneous in clinical characteristics, and the majority did not take into account different treatment modalities in performance evaluation. In this retrospective study, we determined the prognostic value of various CTHA parameters in terms of survival in a large cohort of advanced NSCLC patients and their correlation with genetic alterations. We then validated the outcome in another dataset of advanced NSCLC patients.

## RESULTS

**Figure 1:**
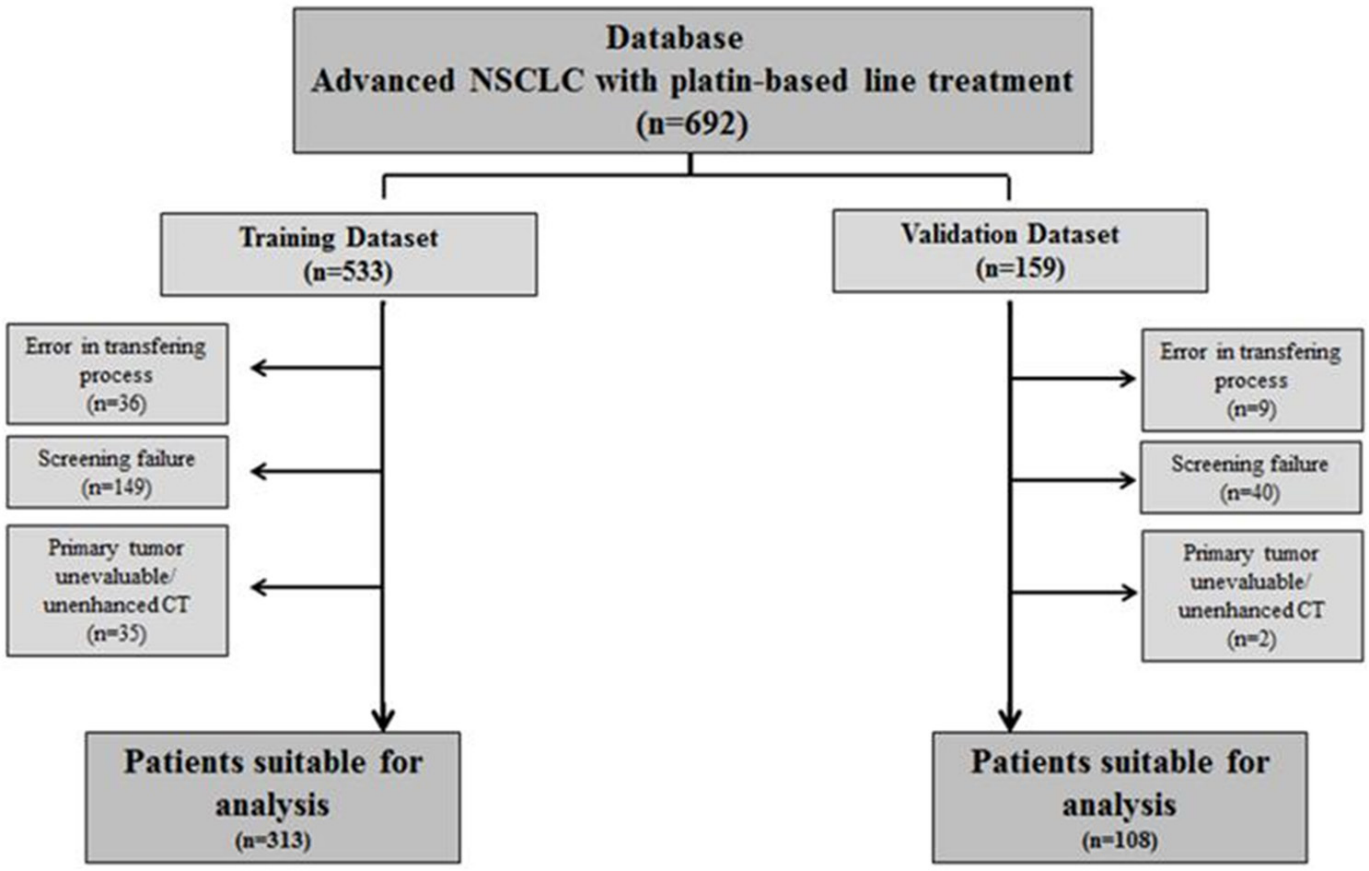
Patient flowchart

Out of 692 patients with contrast-enhanced CT scans screened, 421 (60.8%) were suitable for histogram analysis. Patients were divided into the training dataset (n=313) and the validation dataset (n=108). The main reason for exclusion from the training dataset were screening failure (due to < stage IV at inclusion, absence of platinum-based treatment, other histology than NSCLC and baseline CT not available) followed by errors in the transferring process and non-evaluable primary tumor. Screening failure was also the main reason for exclusion in the validation dataset (Figure 1).

### Patient characteristics

Patient characteristics are shown in Table 1 and were well balanced between the two patient cohorts, with the exception of age, primary size tumor distribution and type of platin-based treatment, however all of the patients have received platin-based chemotherapy for metastatic disease (no difference between carboplatin-based and cisplatin-based chemotherapy in overall survival [HR 1.00, 95%CI: 0.51-1.97, I(2) = 0%] and one-year survival rate [RR 0.98, 95% CI 0.88- 1.09, I(2) = 24%] has been demonstrated within a comparative analysis of both drugs in a recent metaanalysis [17]). There was no difference Molecular status was available for approximately three-quarters of the population; approximately half of the evaluable population had at least one molecular aberration, mainly *KRAS* mutation (20%), *EGFR* mutation (11%) and *ALK* translocation. The median follow-up was 33.7 months [range: 1.7 - 65.5] in the training dataset and 29 months [range: 1.1 - 35.6] in the validation dataset. A total of 252 deaths occurred with a median OS of 11.7 months [95%CI: 10.7 - 13.1] in the training dataset and 84 deaths with median OS of 9.5 months [95%CI: 7.9 - 12.7] in the validation dataset.

**Table 1:** Patient characteristics

	Training dataset n=313 (%)	Validation dataset n=108 (%)	*p*-Value^*^
**Age at diagnosis (years)**			0.006
Median [range]	59 [25 – 83]	63 [30 – 82]	
**Sex**			0.32
Female	124 (40)	37 (34)	
Male	189 (60)	71 (66)	
**Smoking status**			0.16
Never-smoker	50 (16)	11 (10)	
Former/current	263 (84)	95 (90)	
Missing	-	2	
Performance Status^**^			0.82
0-1	247 (81)	85 (82)	
2-3	59 (19)	19 (18)	
Missing	7	4	
**Histology**			0.17
Adenocarcinoma	235 (75)	83 (77)	
Squamous cell	33 (11)	16 (15)	
Other	45 (14)	9 (8)	
**Primary size tumor (cm)**			
Median [range]	33 [10 - 166]	38 [11 – 54]	0.001
≤40	193 (62)	54 (52)	
>40	120 (38)	49 (48)	
Missing	-	5	
**Stage at diagnosis**			0.94
I-II	12 (4)	4 (4)	
III-IV	299 (96)	104 (96)	
Missing	2	-	
**Treatment**			0.010
Carboplatin	143 (46)	34 (31)	
Cisplatin	170 (54)	74 (69)	
**Number mutated/amplified genes**			0.054
0	108 (44)	26 (32)	
1-4	136 (56)	55 (68)	
Unknown	69	27	
**Molecular characterization**	**n=244**	**n=81**	
Wild type	108 (44)	26 (32)	
ALK	15 (6)	3 (4)	
EGFR	26 (11)	7 (9)	
KRAS	49 (20)	12 (15)	
ALK/EGFR	1 (0.4)	-	
ALK/KRAS	2 (0.8)	-	
EGFR/KRAS	1 (0.4)	-	
ALK/Other^*^	2 (0.8)	-	
EGFR/Other^*^	1 (0.4)	1 (1)	
KRAS/Other^*^	11 (5)	10 (12)	
Other^*^	28 (11)	22 (27)	

^*^ *p*-Value was calculated using χ^2^ for categorical variables and student test for continuous variables.

^**^ WHO criteria.

^*^OTHER: HER2, MET, TP53, STK11, CDKN2, PTEN, FGFR2A, FGFR1, KDR, PI3KCA, PDPK1, NRAS.

### HA prognosis markers in the training dataset

Primary mass entropy in coarse texture with spatial filter 3.3 (HR: 1.3 [95%CI: 1.1 - 1.5], *p*=0.001) was significantly associated with OS in the univariate analysis, and there was a trend toward better survival for the coarse texture with spatial filter 2.8 (HR: 1.26 [95%CI: 1.1 - 1.5], *p*=0.007) and medium texture with spatial filter 2.2 (HR: 1.3 [95%CI: 1.1 - 1.6], *p*=0.006). No statistically significant associations were identified for primary mass entropy with the finest textures (spatial filter 1.7: HR: 1.2 [95%CI: 0.9 - 1.5], *p*=0.248; spatial filter 1.1: HR: 1.2 [95%CI: 0.9 - 1.6], *p*=0.143). None of the other parameters analyzed achieved significant associations with OS.

Smoker status (HR: 1.9 [95%CI: 1.3 - 2.7], *p*=0.001), PS (HR: 2.2 [95%CI: 1.6 - 3.0], *p*<0.0001), primary size tumor (HR: 1.01 [95%CI: 0.99 – 1.01], *p*= 0.005), adrenal (HR: 1.9 [95%CI: 1.4-2.5], *p*< 0.0001) and liver metastases (HR: 1.8 [1.3-2.6], *p*= 0.0004), were found significantly associated with poorer outcome in univariate analysis for overall survival. After adjustment by the mentioned prognostic factors, the primary mass entropy in coarse texture with spatial filter 3.3 was still found prognostic for overall survival within the multivariate cox analysis (HR: 1.3 [95%CI: 1.1 - 1.5], *p*=0.002). Summary of the univariate and multivariate analysis for overall survival of the parameter primary mass entropy is exposed in Table 2.

**Table 2:** Univariate and multivariate analysis for overall survival: Prognostic factors and histogram analysis parameters in the training dataset

Prognostic factor/parameter	N events/ N patients	Univariate analysis (n=313)		Multivariate analysis (n=301)	
		HR [95% CI]	*p*-Value (Wald)	HR [95% CI]	*p*-Value (Wald)
**Smoker status**					
**Never**	33/50	1	0.001	1	**0.002**
**Former/current**	219/263	1.9 [1.3-2.7]		1.8 [1.3-2.7]	
**Performance Status (UK=7)**					
**0-1**	194/247	1	<.0001	1	**<0.0001**
**>1**	53/59	2.2 [1.6-3.0]		2.3 [1.7-3.2]	
**Primary size tumor (UK=5)**	248/308	1.01 [1.00-1.01]	0.005	1.0 [0.99-1.01]	0.89
**Adrenal metastasis**					
**No**	186/241	1	<.0001	1	**0.002**
**Yes**	66/72	1.9 [1.4-2.5]		1.6 [1.2-2.2]	
**Liver metastasis**					
**No**	211/269	1	0.0004	1	**0.003**
**Yes**	41/44	1.8 [1.3-2.6]		1.7 [1.2-2.4]	
**Coarse (2.2) (UK=5)**					
**Entropy**	251/312	1.3 [1.1-1.6]	0.006	-	**-**
**Coarse (2.8) (UK=5)**					
**Entropy**	250/311	1.25 [1.1-1.5]	0.007	-	**-**
**Coarse (3.3) (UK=5)**					
**Entropy**	248/308	1.3 [1.1-1.5]	0.001	1.3 [1.1-1.5]	**0.002**

UK: Unknown.

### Correlation with molecular profile in the training dataset

No texture analysis parameters significantly discriminated mutational status for *EGFR* mutated versus non-mutated, *ALK* + versus *ALK* - and *KRAS* mutated versus *KRAS* non-mutated profile.

### Application of HA prognosis markers in the validation dataset

In the validation dataset, primary mass entropy was not significantly associated with OS for any of the textures that were found to be significant in the Training Dataset (Table 3): medium texture with spatial filter 2.2 (HR: 1.4 [95%CI: 0.9 - 2.2], *p*=0.18), coarse texture with spatial filter 2.8 (HR: 1.2 [95%CI: 0.9-1.7], *p*=0.29) or coarse texture with spatial filter 3.3 (HR: 1.2 [95%CI: 0.9-1.6], *p*=0.18) in the univariate analysis, or the multivariate analysis (HR: 1.1 [95%CI: 0.8-1.5], *p*=0.65). Nor were any other parameters identified as significantly associated with survival, although were not planned for this analysis. Furthermore the prognostic factors significantly associated with OS differ from those found in the Training Set. In the validation cohort, female sex (HR: 0.5 [95%CI: 0.3-0.8], *p*=0.004) was the only prognostic factor found to be significantly associated with better outcome in the univariate analysis. The well-known adverse prognostic factors as PS (PS >1 versus ≤ 1 HR: 1.8 [95%CI: 1.0-3.1], *p*=0.04) and smoking status (ever smoker versus never smoker HR: 1.8 [95%CI: 0.8-4.0], *p*=0.12) did not reach statistical significance in this series.

**Table 3:** Univariate and multivariate analysis for overall survival: prognostic factors and histogram analysis parameters in validation dataset

Prognostic factor/parameter	N evt/N pts	Univariate analysis (n=108)		Multivariate analysis (n=99)	
		HR [95% CI]	*p*-Value (Wald)	HR [95% CI]	*p*-Value (Wald)
**Smoker status**					
**Never**	7/11	1		1	
**Former/current**	76/95	1.8 [0.9-4.0]	0.12	2.1 [0.8-5.1]	0.11
**Performance Status**					
**0-1**	65/85	1		1	
**>1**	16/19	1.8 [1.0-3.1]	0.039	1.6 [0.9-2.9]	0.12
**Primary size tumor**	80/103	1.0 [0.99-1.01]	0.70	1.0 [0.99-1.01]	0.97
**Adrenal metastasis**					
**No**	49/91	1	0.21	1	0.01
**Yes**	11/17	1.5 [0.8-2.9]		2.2 [1.2-4.0]	
**Liver metastasis**					
**No**	51/93	1	0.38	1	0.67
**Yes**	9/15	1.4 [0.7-2.8]		1.2 [0.6-2.2]	
**Coarse (2.2)**					
**Entropy**	84/108	1.2 [0.7-2.1]	0.59	-	-
**Coarse (2.8)**					
**Entropy**	84/108	1.2 [0.8-1.8]	0.37	-	-
**Coarse (3.3)**					
**Entropy**	84/108	1.2 [0.9 - 1.6]	0.18	1.1 [0.8-1.5]	0.65

As was the case in the Training Dataset, no histogram analysis parameters significantly discriminated the mutational status for the *EGFR*-mutated versus non-mutated, *ALK*+ versus *ALK*- and *KRAS* mutated versus non-mutated profiles.

## DISCUSSION

This study aimed to determine the potential of imaging analysis as an independent predictor of survival and the potential to associate tumor heterogeneity with gene alterations in patients with NSCLC. In the training dataset primary mass entropy was significantly associated with OS in the univariate analysis and remained an independent prognostic factor in a multivariate analysis. This finding is coherent with two published studies in early-stage and locally-advanced NSCLC in which primary mass entropy was reported to be prognostic for OS [16, 18]. The prospective application of significant parameters in the validation dataset was intended to increase statistical robustness. However we were unable to validate the prognostic value of primary mass entropy. A literature search revealed only one study using an independent validation dataset cohort, however different parameters were analyzed between sets, limiting the validity of their results [19]. Several studies have reported different pairs of positive significant associations between different parameters and survival in NSCLC. A systematic review conducted by Chalkidou *et al* in 2015, highlighted the lack of agreement between parameters identified in published studies and even contradictory results for the same features [20]. To date, the limited evidence available relies on retrospective series of heterogeneous population with small sample sizes and is insufficient to support a correlation between CT features and survival. To our knowledge, our cohort represents the largest series evaluating the prognostic performance of histogram analysis features in advanced NSCLC.

We did not find an association between entropy and molecular characteristics (*KRAS* and *EGFR* mutation, *ALK* rearrangement). Furthermore, no single parameter significantly correlated with any of the three most frequent mutations analyzed. The fact that skewness was not significantly associated with a given molecular profile led us to question our earlier findings over the *EGFR* mutated/*ALK* rearranged cohort. However, differences concerning populations and type of analysis between both studies, made expected results difficult to compare [21]. Several studies in the past decade have emphasized on lung cancer's molecular heterogeneity [22]; histogram analysis may reflect a pattern or group of mutations, but from our perspective it is overly simplistic to correlate a single parameter in order to establish a definitive and reliable conclusion.

Our study has a number of limitations. First, as a retrospective study, the potential for selection biases (e.g. restricting the analysis population to primary tumor) and differences concerning settings and protocol CT acquisitions cannot be excluded; histogram analysis assessed on contrast-enhanced CT scans can be affected by several variables, such as interscanner variability, pixel values, imaging parameters and contrast media injection rate [23, 24]. The impact of this variability on HA features was not evaluated in this study; prospective studies are needed to measure the true impact of HA features. Patient variables (e.g., cardiac output and body mass index) may influence tumor enhancement. In addition, only a single largest cross section was used for analyses, which may not have been representative of tumor heterogeneity [25].

The inability to reproduce primary mass entropy as an independent prognostic factor in the validation patient cohort may have been due to the smaller size of the validation dataset, although statistical power was adequate to detect a reasonable effect size. Few publications address the possibility of false discovery rates in image analysis despite that investigation of multiple variables in single datasets may increase this phenomenon [26]. Although the analysis of large quantities of variables may have increased the risk of false positives findings, we decreased this risk by strictly adjusting the *p-*value cut-off (p<0.005); nonetheless, our validation results did not confirm the ones from training dataset. Verifying outcomes within a properly selected prospective validation dataset should help clarify this issue. Variations within the tumor (necrosis, angiogenesis, hypoxia, etc.) at a cellular level may potentially be translated into variations within pixel distribution, however this must be confirmed with a software that integrates other aspects of the tumor images such as shape, wavelet among others, more uniform CT scan acquisition and large patient cohorts before it can be implemented into routine clinical practice.

## MATERIALS AND METHODS

### Patients

The overall study population consisted of 692 patients with advanced NSCLC (stage IIIB not amenable to loco-regional treatment or stage IV) included in the MSN-LUNG study (CSET: 2007/1363) who received platinum-based chemotherapy at Gustave Roussy (France) between July 2009 and January 2015. All patients had to have a diagnostic-quality contrast-enhanced CT scan with an evaluable primary tumor, acquired for diagnostic/staging routine. Molecular profiling was performed for the MSN study over patient's archived diagnostic tissue. The MSN study was approved by the institutional ethics committee board and all patients signed an inform consent for tumor analysis. CT scans, clinical and molecular data (*KRAS*, *EGFR* and *ALK* status) were retrospectively collected for correlation with HA parameters. Two datasets were designated, 1) a training dataset to derive optimal HA parameters for predicting survival and molecular profile, and 2) a validation dataset to prospectively apply these findings. The training dataset was composed of patients included in the MSN-LUNG study from July 2009 to December 2013, and the validation dataset was composed of patients included from December 2013 to January 2015

### CT acquisition

CT scans from routine diagnosis or staging of lung cancer were retrospectively reviewed. Scans were performed in different centers, using various devices and protocols for acquisition parameters (kVp, mAs, slice thickness, pitch, and reconstruction algorithm). A diagnostic-quality contrast-enhanced CT scan with an evaluable primary tumor was required, acquired for diagnostic/staging for locally advanced disease or within two weeks after initiating systemic treatment for advanced disease at diagnosis. Scans without contrast injection were excluded. CT scans without evaluable primary tumor due to primary surgery, pleural effusion, previous chest radiotherapy, or with indistinguishable tumor margin from combined atelectasia or adjacent nodal structure were excluded.

### Histogram analysis

CTHA was conducted by an oncologist supervised by a senior radiologist using proprietary software developed by Ganeshan *et al* (TexRAD) [7].

### Identification and delineation of the ROI

The primary lung tumor image or the image with the largest cross-sectional area when more than one image was available, were selected for delineating the region of interest (ROI). The ROI was drawn manually around the entire tumor boundary in a single 2-5 mm axial slice using a semi-automated process and used for image filtration and histogram quantification [7].

### Image filtration and texture quantification

Computed tomography texture analysis involved an initial image filtration step using Laplacian of Gaussian spatial bandpass filters at a range of spatial frequencies followed by texture quantification in the filtered images. These filters were each applied to the selected ROI, producing filtered images that highlighted features of a specific size at different spatial periods (1/spatial frequency) from 2 mm (fine texture) to 6 mm (coarse texture). These filters were termed spatial scale filters (SSFs). The filtered conventional CT images were SSF corresponds to fine to coarse filtered texture (enhancing objects of 2-mm radius to 6 mm radius). The filter sigma represents the spatial frequencies that comprise the image filtrations, with a sigma scale to extract features of fine, medium and coarse texture within fine (SSF 2: sigma median 1.1), medium (SSF3, sigma median 1.7, SSF 4: sigma median 2.8) and coarse texture (SSF 5: sigma median 2.8, SSF 6: sigma median 3.3) [7]. The histograms of the pixel values in the filtered images were quantified using standard descriptors: mean, standard deviation (SD), skewness, kurtosis, entropy and mean of positive pixels (MPP) [9]. First-order statistics were based on the probability distribution of individual grey-level pixel values (mean, entropy-irregularity, uniformity-homogeneity), second-order were based on the joint probability distributions of pairs of pixels (SD-histogram variance), third-order on skewness-asymmetry of the histogram, and fourth-order on kurtosis- pointiness [7].

### Statistical analysis

The primary objective was the assessment of the prognostic value of CTHA in a cohort of advanced NSCLC patients treated with platinum-based chemotherapy in terms of overall survival (OS). The secondary objective was the correlation of CTHA parameters with gene alterations. Statistical analyses were performed using SAS version 9.3 (SAS institute INC. Cary NC, USA).

OS was defined from the first platinum-line treatment to death from any cause or the last follow-up and curves were estimated with the Kaplan-Meier method. The prognostic value of the following covariates was measured in a univariate analysis: sex, age at inclusion (>70 years-old), smoker status (never/passive smoker versus ex/current smoker), performance status (PS) (≤1 versus >1), primary size tumor (≤ vs. > 40 cm), presence of adrenal and liver metastases and CTHA parameters. Multivariate analysis was performed by Cox regression models, forcing the clinical factors in the model and adding separately the candidate radiomics features (one Cox model by feature). The Wilcoxon test was used to correlate CTHA parameters with mutational status (*EGFR*, *ALK* and *KRAS*). Because of strong dependence between the different measures across filter values we a priori prespecified a p-value cutoff of 0.005 to declare significance in the training series to adjust for multiple testing.

## CONCLUSION

The potential for exploiting translation of heterogeneity via histogram analysis for predicting outcome was not confirmed. The apparent lack of validity of these techniques raises questions over their relevance and the quality of data reported to date.
